# Combining the oncolytic peptide LTX-315 with doxorubicin demonstrates therapeutic potential in a triple-negative breast cancer model

**DOI:** 10.1186/s13058-018-1092-x

**Published:** 2019-01-22

**Authors:** Ketil A. Camilio, Meng-Yu Wang, Brynjar Mauseth, Stein Waagene, Gunnar Kvalheim, Øystein Rekdal, Baldur Sveinbjørnsson, Gunhild M. Mælandsmo

**Affiliations:** 1 Department of Tumor Biology, Institute for Cancer Research, Oslo University Hospital, NO-0379 Oslo, Norway; 2 Department of Cellular Therapy, Oslo University Hospital, NO-0379 Oslo, Norway; 3 Lytix Biopharma AS, Hoffsveien 4, NO-0275 Oslo, Norway; 4 Department of Medical Biology, The Arctic University of Norway, NO-9037 Tromsø, Norway; 5 Division of Cancer, Surgery and Transplantation, Oslo University Hospital, Rikshospitalet, NO-0372 Oslo, Norway; 6 Institute of Clinical Medicine, University of Oslo, NO-0372 Oslo, Norway; 7 Oslo Cancer Cluster Incubator, Ullernchausseen 64/66, 0379 Oslo, Norway

**Keywords:** Triple-negative breast cancer, Immunochemotherapy, Combination therapy, Immunotherapy, Oncolytic peptide, LTX-315, Doxorubicin, DAMPs, ICD

## Abstract

**Background:**

Immunochemotherapy, the combined use of immunotherapy and chemotherapy, has demonstrated great promise in several cancers. LTX-315 is an oncolytic peptide with potent immunomodulatory properties designed for the local treatment of solid tumors. By inducing rapid immunogenic cell death through the release of danger-associated molecular pattern molecules (DAMPs), LTX-315 is capable of reshaping the tumor microenvironment, turning “cold” tumors “hot” through a significant increase in tumor-infiltrating lymphocytes.

**Methods:**

We investigated the potential of LTX-315 to be used in combination with standard-of-care chemotherapy (doxorubicin, brand name CAELYX®) against triple-negative breast cancer in an orthotopic 4 T1 mammary fat pad model. Tumor growth curves were compared using one-way ANOVA analysis of variance and Tukey’s multiple comparisons test, and animal survival curves were compared using the log-rank (Mantel-Cox) test. We considered *p* values ≤0.05 to indicate statistical significance.

**Results:**

We found that LTX-315 displayed a strong additive antitumoral effect when used in combination with CAELYX®, and induced immune-mediated changes in the tumor microenvironment, followed by complete regression in the majority of animals treated. Furthermore, imaging techniques and histological examination showed that the combination induced strong local necrosis, followed by an increase in the infiltration of CD4+ and CD8+ immune cells into the tumor parenchymal tissue.

**Conclusions:**

Our data demonstrate that LTX-315 is a promising combination partner with CAELYX® for the treatment of triple-negative breast cancer.

## Background

Breast cancer remains one of the four major cancers, and is expected to account for approximately 30% of all new cancer diagnoses in women in the USA, making it the leading cause of cancer-related deaths in women aged 20–59 years [1]. Triple-negative breast cancer (TNBC) represents 15% of breast carcinomas, a particularly aggressive and heterogeneous subtype defined by the absence of the three primary breast cancer biomarkers - estrogen receptor (ER), progesterone receptor (PR) and human epidermal growth factor receptor 2 (HER2, also known as ERBB2). Triple-negative breast cancer is recognized by high proliferative activity, increased immune cell infiltration, and a more basal-like and mesenchymal phenotype. Patients with TNBC do not benefit from endocrine or anti-HER2 therapy, leaving chemotherapy as the only established option [2]. Resistance usually develops after an initial period of response [3]. Hence, there is an urgent need for innovative treatment options that can improve disease outcome or complement existing therapies for patients with breast cancer. With the introduction of immune-based therapies, such as checkpoint blockade, significant advances have been made in the treatment of cancer, thereby illustrating the importance of harnessing the immune system. However, immunotherapy has shown limited success in the treatment of breast cancer [4]. The next step to further increase the clinical benefit of breast cancer therapy will be to target the antitumor immune response at multiple levels, which may be accomplished through combination therapy approaches. Turning cold tumors hot would further augment the antitumor efficacy of established therapies such as checkpoint blockade [5]. By using immune-based combination therapy it is possible to shift the clinical response to a durable response due to immunological memory [6].

Host defense peptides, commonly denoted as cationic antimicrobial peptides (CAPs) [7], often possess anticancer and immune-modulating properties due to their amphipathic nature and cationic charge. Several CAPs have been shown to target cancer cells by interacting with and destabilizing anionic lipid membranes or highly negatively charged intracellular targets such as mitochondria [8], thus emphasizing their potential as novel anticancer agents [9–15]. We have previously described the development of LTX-315, a first-in-class oncolytic peptide used for intratumoral treatment of solid tumors [16, 17]. The mode of action is associated with a reshaping of the tumor microenvironment, in which LTX-315 induces immunogenic cell death through a membranolytic effect (necrosis), as well as direct effects on intracellular targets such as mitochondria [18–21]. Changes to the tumor microenvironment are dependent on the release of potent danger-associated molecular pattern molecules (DAMPs) and tumor antigens that recruit and activate dendritic cells with subsequent T-cell activation and tumor-specific immune responses. Intratumoral treatment with LTX-315 results in growth inhibition, complete regression and long-lasting tumor-specific immune responses in a variety of different experimental animal models [22–24].

Conventionally, chemotherapeutic agents have been used in the clinic based on their capacity to directly kill proliferating cells. However, although high-dose chemotherapy has been shown to significantly debulk the tumor, it often leads to disease relapse and the establishment of drug resistance. There is now a growing focus on a new therapeutic paradigm using low-dose or medium-dose chemotherapeutics at short repeated intervals due to their potential to stimulate anticancer immune responses while selectively eliminating immunosuppressive cells [25]. For this reason, chemotherapy may be a promising candidate in combination with immunotherapy, defined as immunochemotherapy, as shown by augmented anticancer effects following treatment with chemotherapy and anticancer vaccines [26, 27]. Furthermore, doxorubicin has been shown to induce immunogenic cell death in addition to inhibit suppressive cells such as myeloid-derived suppressor cells, thereby removing the brakes implemented on the immune system by such cells [28–30].

In the present study, we investigated the potential of combining intratumoral treatment with LTX-315 and CAELYX® (liposomal doxorubicin) in a preclinical TNBC model. The unique mode of action of LTX-315, reprogramming and reshaping the tumor microenvironment, thus turning “cold” tumors into “hot” tumors, showed a significant additive antitumor effect when combined with CAELYX®. It is well-established that tumor-infiltrating CD8^+^ T cells are important for therapeutic efficacy, and help predict clinical outcome in breast cancer [5, 31]. LTX-315 induced an increase in the infiltration of cytotoxic CD8^+^ T cells into the viable tumor bed, creating an immunogenic antitumor microenvironment. Combining LTX-315 with doxorubicin further augmented this effect.

## Methods

### Aim and study design

**Fig. 1:**
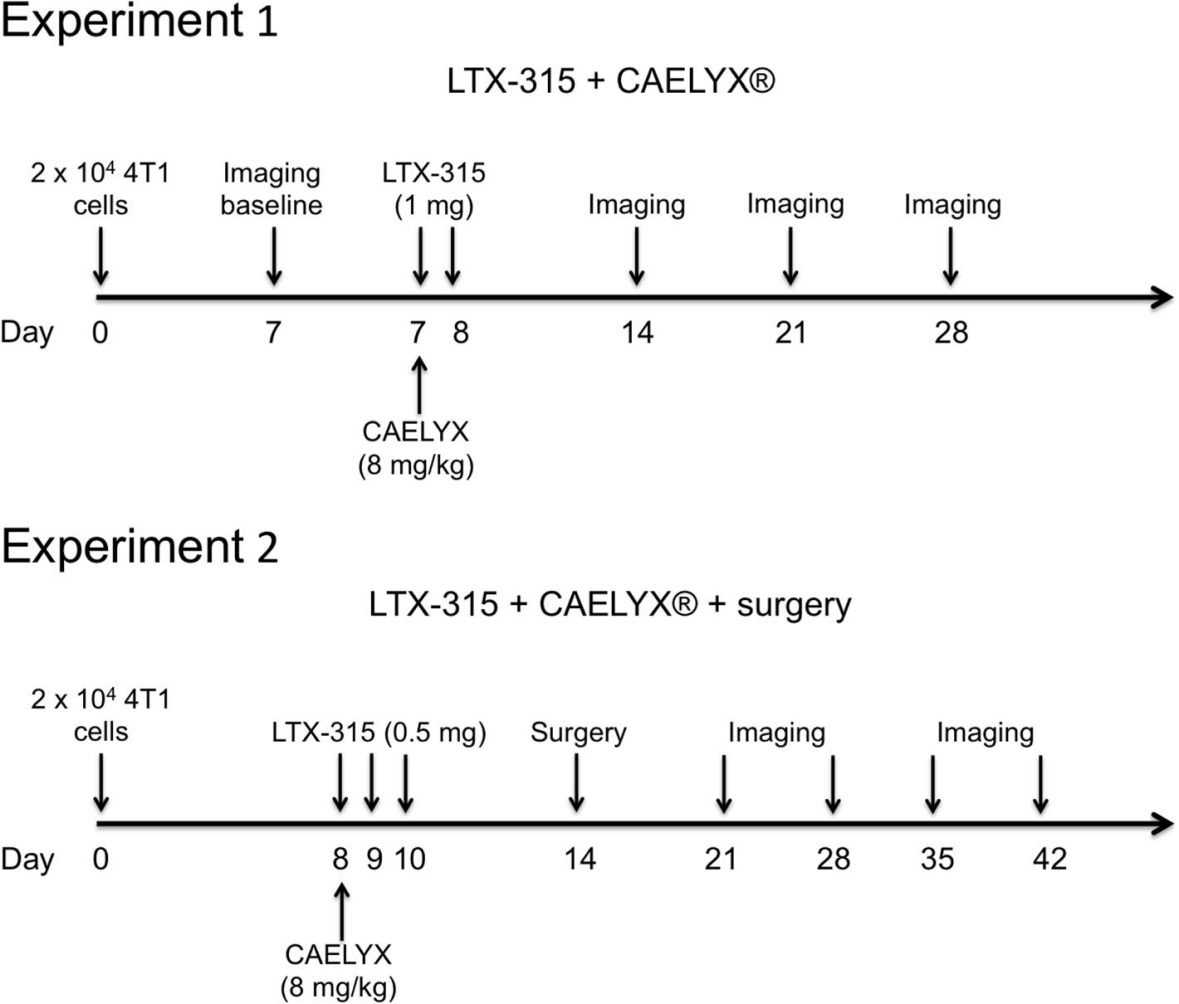
Experimental protocols. Freshly harvested 4 T1 tumor cells were injected into the mammary fat pads of syngeneic (Balb/C) mice. One week after transplantation, the mice were randomized into four treatment groups: (i) vehicle (saline), (ii) CAELYX®, (iii) LTX-315, and (iv) LTX-315 + CAELYX® for experiment 1 and (i) vehicle (saline) + surgery, (ii) CAELYX® + surgery, (iii) LTX-315 + surgery, and (iv) LTX-315 + CAELYX® + surgery for experiment 2

The aim of this study was to investigate the antitumor efficacy of LTX-315 in combination with CAELYX® in a preclinical TNBC animal model (experiment 1, Fig. 1). Additive antitumor effects were monitored by tumor size, imaging, histology and survival. Experiment 2 was designed to mimic the clinical situation, and evaluate whether larger 4 T1 tumors could be eradicated in a neoadjuvant setting, combining LTX-315 and CAELYX® with surgery (experiment 2, Fig. 1).

### Cell lines and reagents

The 4 T1 (ATCC, CRL-2539), a murine triple-negative breast cancer cell line was purchased from the American Type Culture Collection (ATCC-LGC Standards, Rockville, MD, USA). The 4 T1 cell line is used as an animal model for stage IV human breast cancer. The cells were transduced using pCDH-EF1α-extGlucT2A-mKate-NGFR (gift of Dr Irmela Jeremias Helmholtz Zentrum München, Munich, Germany). Third-generation packaging plasmids pMDLg/pRRE, pRSV-Rev, and pMD2-G (Addgene, Teddington, UK) were used. High-titer vesicular stomatitis virus (VSV) G-protein-pseudotyped lentivector was prepared by transient 4-plasmid transfection of 293 T cells using a TurboFect Transfection Reagent (Thermo Scientific, Waltham, MA, USA). LTX-315 (K-K-W-W-K-K-W-Dip-K-NH_2_) was produced and purchased on request from Bachem AG (Bubendorf, Switzerland). CAELYX® (pegylated liposomal doxorubicin hydrochloride, 2 mg/ml) was purchased from the Hospital Pharmacy at Oslo University Hospital – Radiumhospitalet.

### Animals

Female Balb/C wild-type mice, 5–6 weeks old, were obtained from Janvier Labs, Le Genest-Saint-Isle, France. During the experiments, mice weighing 18–25 g were kept in groups of 8–10 animals per cage under climate-controlled conditions, with 12-h light/dark cycles and an ambient temperature. Animals were housed in suitable cages with free access to standard rodent chow and water *ad libitum*. The animals were anesthetized during the experimental procedures with 5% sevofluran as an induction dose and 3–4% as a maintenance dose during imaging or surgery, which provided a sufficient degree of sedation and analgesia. The animals were monitored daily and tumor-bearing mice with large tumors were euthanized with cervical dislocation. All procedures performed were conducted under FOTSid 7214 and 9458, and approved by the Experimental Animal Board under the Ministry of Agriculture of Norway and in compliance with The European Convention for the Protection of Vertebrate Animals used for Experimental and other Scientific Purposes. The laboratory animal facilities are subjected to a routine health-monitoring program, and were screened for common pathogens according to a modification of the Federation of European Laboratory Animal Science Association’s recommendation.

### Tumor challenge

Tumor cells were harvested, washed in PBS and injected into the 4th inguinal mammary fat pad on the right-hand side of syngeneic recipient mice as follows. A small incision was made to the ventral skin using a scalpel, and the skin was deflected using suitable surgical instruments to access the mammary fat pad. The mammary fat pad was then fixated using Kelly forceps and injected with 2 × 10^4^ 4 T1 cells per mouse/20 μl PBS using Braun Omnican 50 insulin syringes (VWR International AS, Oslo, Norway). Finally, the surgical incision was closed using Polysorb™ braided absorbable suture 5–0 from Medtronic (Oslo, Norway) and Histoacryl® biological glue from B. Braun (Melsungen, Germany). The tumor volume was measured using an electronic caliper and expressed as volume V = (L × W × W)/2, where V is the tumor volume, W is the tumor width and L is the tumor length. The animals were euthanized when the tumor volume reached 1600 mm^3^ or when tumor ulceration and/or severe metastasis developed.

### Peptide treatment

Palpable tumors (40–80 mm^3^ on day 7 or 8) were injected intratumorally with single doses of LTX-315 in saline (0.5–1.0 mg peptide/50 μl saline) once a day for 2–3 consecutive days (see Fig. 1). The vehicle control was saline only (0.9% NaCl in sterile H_2_O).

### Chemotherapy treatment

Treatment with CAELYX® was initiated on the same day as LTX-315 treatment (see Fig. 1). Animals with palpable tumors (40–80 mm^3^ on day 7 or 8) were injected intravenously with a single dose of CAELYX® (8 mg/kg) in a volume of 200 μl.

### Neoadjuvant treatment and surgery

Animals with larger tumors (60–100 mm^3^ on day 8) were treated as previously described and anesthetized to surgically excise tumors on day 14 (day 6 post-treatment). Following a small incision to the skin the tumor was removed using suitable surgical equipment. All blood vessels were closed using a BOVIE Disposable Cautery Pen (Agntho’s AB, Lidingö, Sweden). Last, the surgical incision was closed using Polysorb™, a braided absorbable suture 5–0 from Medtronic (Oslo, Norway) and Histoacryl® biological glue from B. Braun (Melsungen, Germany). Animals that underwent surgery were given Temgesic (from the Hospital Pharmacy at Oslo University Hospital – Radiumhospitalet) subcutaneously (s.c.) twice per day at 8-h intervals (100 μl using a stock solution of 0.3 mg/ml diluted 1:10 in sterile H_2_O).

### Bioluminescence imaging (BLI)

For bioluminescence in vivo imaging mice were anesthetized with sevoflurane as previously described. Tumor growth was monitored using a field of view of 12.5 cm with binning 8, f/stop 1 and an open filter setting using the IVIS Spectrum In Vivo Imaging System (Perkin Elmer, MA, USA). Mice were injected with 200 μl/animal D-Luciferin (20 mg/ml; Biosynth, Staad, Switzerland) intraperitoneally 10 min before imaging was initiated and ventral images were taken. Images were taken once weekly, starting at treatment baseline (day 7 or 8). The imaging data were then analyzed using the Living Image software (Perkin Elmer, MA, USA), as radiance photons/sec/cm^2^/steradian (p/sec/cm^2^/sr).

### Magnetic resonance imaging (MRI)

MRI was performed using a 7 T MR system (Bruker BioSpin MRI GmbH, Ettlingen, Germany, software ParaVision 6.0). Three animals from each group were anesthetized with sevofluran and scanned. The animals were placed in the prone position in the scanner, with an abdominal pressure-sensitive probe and a rectal temperature probe (both Small Animal Instruments, Inc., Stony Brook, NY, USA) to monitor respiration rate and temperature. This was connected to the Model 1030 Monitoring & Gating System (Small Animal Instruments, Inc.). The body core temperature of the mice was kept at approximately 37 °C using a fan module (MR-compatible Small Rodent Heater System, Small Animal Instruments, Inc.) adjusting the hot air flow automatically to keep the temperature stable. To help ensure the correct placement of the mice and to localize the tumor, a fast gradient echo localizer scan was performed. For volume measurements, an axial T2-weighted fast spin echo sequence (repetition time (TR) = 3000 ms, echo time (TE) =31.05 ms, field of view = 30 × 30 mm^2^, image matrix = 256 × 256, slice thickness = 0.5 mm, and slice spacing = 0.2 mm), using a mouse quadrature volume coil, was used. The whole tumor volume was covered using 10–12 slices, with the slice representing the largest diameter of the tumor chosen for representative images. All images were analyzed using OsiriX Lite v.8.0.1 software from Pixmeo SARL, Switzerland.

### Histology/immunohistochemistry

Formalin-fixed and paraffin-embedded tissue sections were deparaffinized in Neo-Clear and graded alcohols, hydrated, and washed in PBS. After antigen retrieval in a sodium citrate buffer (pH 6) in a microwave oven, the endogenous peroxidase was blocked by 0.3% H_2_O_2_ for 15 min. Sections were incubated overnight at 4 °C with primary antibody, rabbit monoclonal anti-CD4 (clone ab183685, Abcam) or rabbit monoclonal anti-CD8 (clone ab209775, Abcam). As a secondary antibody, the anti-rabbit-horseradish peroxidase SignalStain® Boost IHC Detection Reagent (Cell Signaling Technology) from Dako was used. A matched isotype control was used as a control for nonspecific background staining.

### Flow cytometry

Tumors were surgically excised on day 7 post treatment following a single intratumoral injection of LTX-315, a single dose of CAELYX® given intravenously, or the combination of both. Tumor tissue was processed by cutting it into small pieces with scissors before being digested in 2.35 ml RPMI-1640 containing 100 μl Enzyme D, 50 μl Enzyme R, and 12.5 μl of Enzyme A, at 37 °C for 90 min according to the manufacturer’s instructions (mouse tumor dissociation kit from MACS Miltenyi Biotec). The enzymatic reaction was terminated by the addition of excess PBS before single-cell suspensions were prepared by passaging the mixture through 70-μm nylon cell strainers (BD Falcon, VWR, Norway). After washing, cells were stained with a live/dead marker using the LIVE/DEAD™ Fixable Yellow Dead Cell Stain Kit (Thermofisher Scientific, USA) before blocking the FcγIII (CD16) FcγII (CD32) receptors to prevent unspecific binding using the Mouse BD Fc Block™ (clone 2.4 G2, BD Biosciences Europe, Norway). Following Fc blockade surface staining was performed for CD45 allophycocyanin (APC) (clone 30-F11), CD3 phycoerythrin (PE) (clone 145-2C11), CD4 PerCP-Cy 5.5 (clone RM4–5) and CD8a fluorescein isothiocyanate (FITC) (clone 53–6.7) (all from BD Biosciences Europe, Norway) at 4 °C for 25 min. Data were acquired using a FACSCanto II flow cytometer (BD Biosciences) with the BD Biosciences FACSDiva Software (version 8.0.1) and analyzed using FlowJo Software (version vX.0.7).

### Statistical analysis

Tumor growth curves were compared using one-way analysis of variance (ANOVA) and Tukey’s multiple comparisons test, and animal survival curves (Kaplan–Meier plot) were compared using the log-rank (Mantel–Cox) test. Flow data were analyzed using a Student’s *t* test. We considered *p* values ≤0.05 to indicate statistical significance.

## Results

### LTX-315 shows a significant additive antitumor effect against 4 T1 mammary carcinomas when combined with CAELYX®

**Fig. 2:**
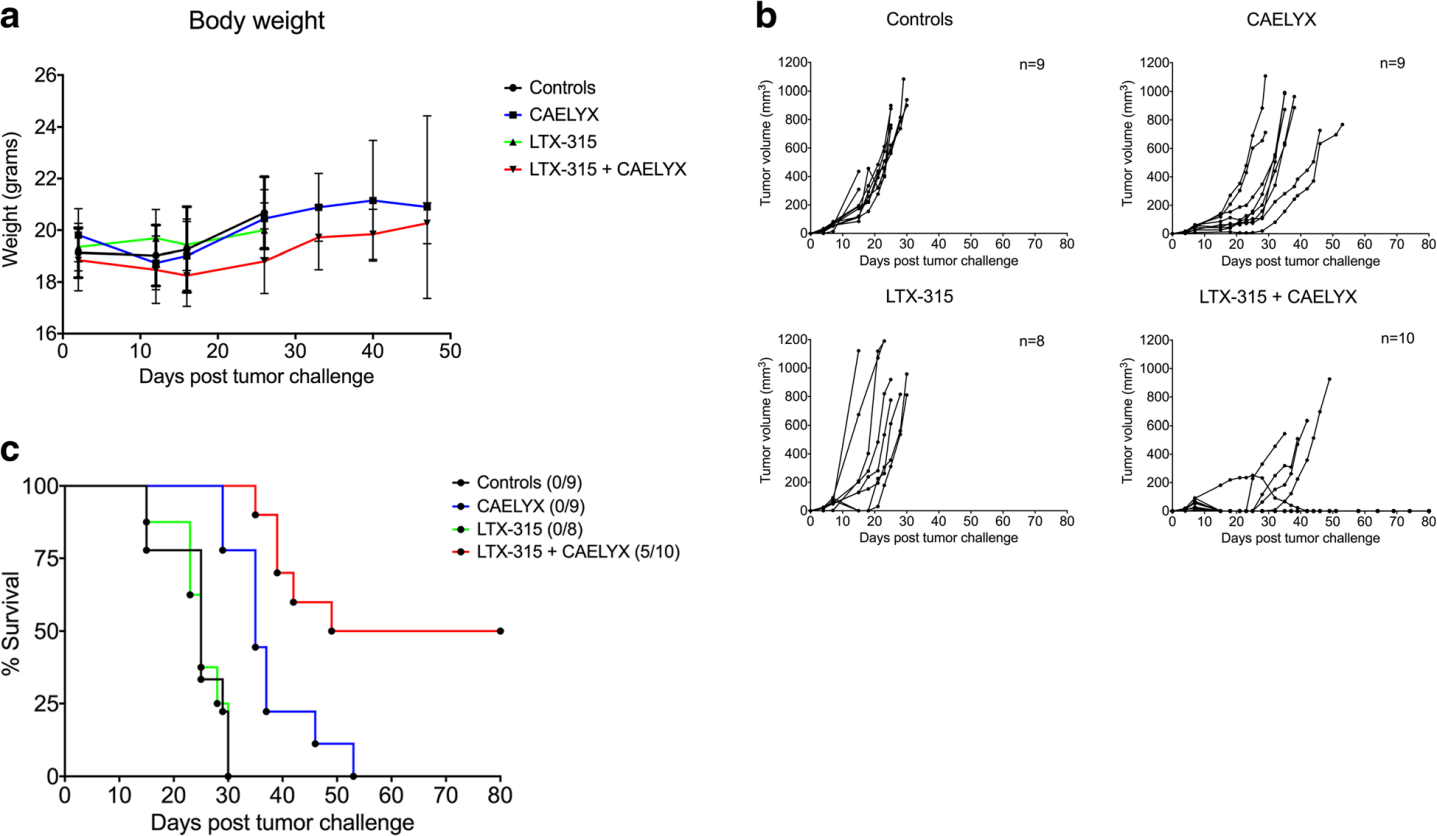
Local treatment with LTX-315 shows a significant additive antitumor effect when combined with CAELYX®. Animals with established 4 T1 mammary carcinomas were divided into four groups, (i) vehicle controls, (ii) CAELYX®, (iii) LTX-315, or (iv) a combination of LTX-315 and CAELYX®. Palpable tumors were injected twice intratumorally with 1 mg LTX-315 on day 7 and 8 post tumor challenge and 8 mg/kg CAELYX® on day 7. The body weight of all animals remained within the normal range throughout the study (**a**). Tumor growth curves showed that LTX-315 had a significant additive antitumor effect (Tukey’s multiple comparisons test, *p* ≤ 0.05) when combined with single-dose CAELYX® compared to monotherapy alone (**b**), in addition to inducing complete regression in 50% of the treated animals (**c**), as shown by the Kaplan–Meier survival plot (*p* < 0.0001)

Animals with palpable 4 T1 mammary carcinomas (40–60 mm^3^) were treated twice intratumorally with LTX-315 (1 mg peptide/injection), in combination with a single dose of CAELYX® intravenously (8 mg/kg body weight). Animals did not display any significant decrease in body weight in the combination group compared to either monotherapy alone, thereby indicating no major increase in adverse events (Fig. 2a). Tumor growth curves demonstrated slight initial growth inhibition after treatment with CAELYX® alone. However, the tumors relapsed shortly thereafter and the animals had to be euthanized due to their tumor burden around day 40–50. LTX-315 monotherapy had no significant impact on tumor growth due to the sub-therapeutic treatment regime used in this study. In highly aggressive tumor models, such as the 4 T1 model, the optimal treatment regime is numerous consecutive injections (three to five) to ensure tumor control and debulking. Interestingly, the combination of LTX-315 and CAELYX® had an additive effect, as there was significant tumor growth inhibition and complete regression in 50% of the treated animals (Fig. 2b and c). Statistical analysis (Tukey’s multiple comparisons test) revealed the combination treatment to exhibit significant additive antitumor effects compared to either monotherapy (*p* ≤ 0.05). The median survival was 25 days in the control and LTX-315 group, 35 days in the CAELYX® group, and 64.5 days in animals treated with the combination approach.

**Fig. 3:**
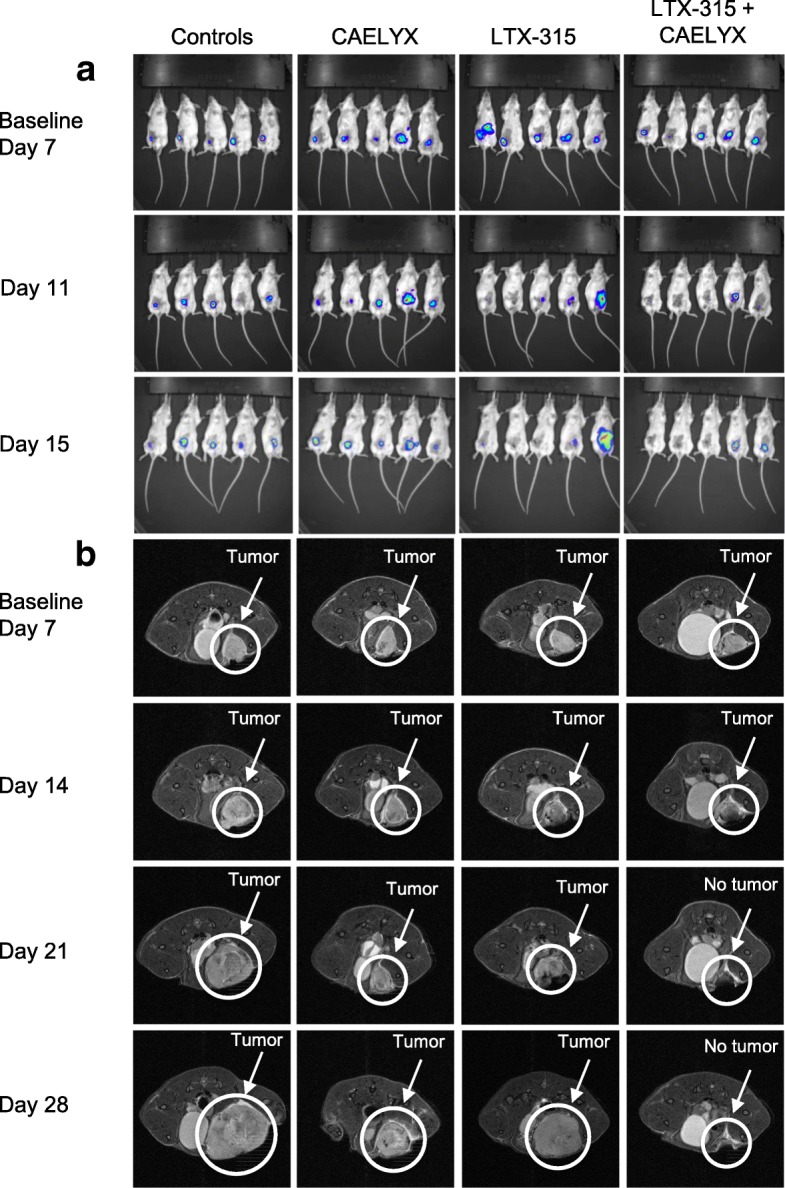
LTX-315 in combination with single-dose CAELYX® induced complete regression of tumors in the majority of animals. Representative bioluminescent (**a**) and magnetic resonance (**b**) images from animals treated with vehicle, CAELYX® alone, LTX-315 alone, or LTX-315 in combination with CAELYX®. Baseline was defined as day 7, and the study was performed as illustrated in experiment 1 in Fig. 1. Animals were scanned at the regular interval of once per week

Animals from the different treatment groups were analyzed for tumor growth, using BLI and MRI (Fig. 3a and b, respectively). As shown by the MRI, control tumors grew rapidly until the tumor burden endpoint (days ~ 20–30). Animals treated with CAELYX® alone experienced tumor growth inhibition for ~ 2 weeks post treatment (day 21) before 4 T1 tumors relapsed and continued to proliferate. The sub-therapeutic intratumoral treatment with LTX-315 induced significant tumor necrosis and debulking of the tumor. Even so, residual breast cancer cells continued to grow and the disease relapsed. The combination of LTX-315 and CAELYX® induced strong debulking of the tumor, thus resulting in significant tumor growth inhibition and complete regression in 50% of the animals, as seen by the images in Fig. 3.

### Combination treatment induces necrosis and T cell infiltration

**Fig. 4:**
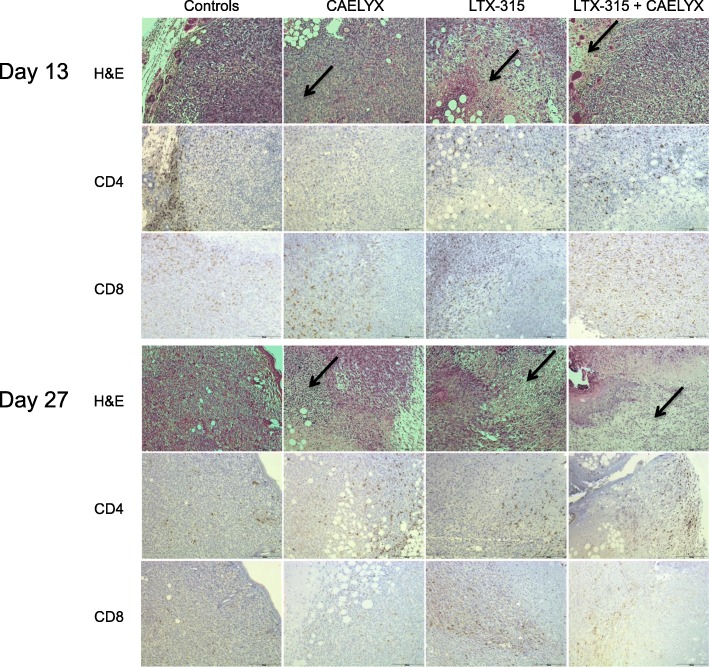
Combination treatment with LTX-315 and CAELYX® induced extensive necrosis and tumor tissue damage followed by an increase in CD8+ tumor-infiltrating T cells into the viable tumor region. Tumors (*n* = 4–5) were harvested on day 13 and 27, and stained for H&E, CD4+ and CD8+ T cells. Representative histological pictures show that tumors treated with monotherapies or the combination therapy had areas of significant necrosis and hemorrhagic damage (black arrows). Treated tumors with remaining viable tumor tissue had significant infiltration of CD4+ T cells compared to tumors in controls in which CD4+ T cells were excluded from the intratumoral milieu. The combination group had higher amounts of CD8+ T cells in the viable intratumoral environment on day 13 compared to controls

Tumors from all treatment groups (*n* = 4) were harvested for histological examination on day 6 post treatment (day 13) and on day 20 post treatment (day 27). Tumors were stained for infiltration of CD3+ (data not shown), CD4+ and CD8+ cells into the peritumoral or intratumoral environment following treatment. Representative images showed that control tumors were viable and proliferating on day 13 and 27. Tumors treated with CAELYX® alone and LTX-315 alone had areas of significant necrosis and hemorrhagic damage at both time points (Fig. 4, top panel, black arrows). LTX-315 monotherapy induced significantly more necrosis and tumor tissue damage compared to CAELYX® alone. However, there was viable tumor tissue with both monotherapies on day 13 and 27. The combination treatment induced massive necrosis and tumor debulking, hence resulting in little or no signs of viable tumor tissue on day 27 (Fig. 4). Control tumors showed that CD4+ T cells were excluded from the tumor parenchyma on day 13 with low infiltration into the viable tumor tissue on day 27. CAELYX® treatment alone resulted in a decrease in the CD4+ population on day 13, before regrowth of the tumor and a significant increase in the CD4+ population on day 27. There was significantly greater intratumoral infiltration of CD4+ T cells in the LTX-315 alone and combination group on day 13 compared to CAELYX® alone and the control group. The biggest difference was observed in the amount of tumor-infiltrating CD8+ T cells. There were low to moderate amounts of intratumoral CD8+ T cells in the viable tumor tissue in the control group and monotherapy groups. Significantly larger amounts were observed in the combination group in the remaining viable tumor tissue on day 13, indicating reshaping of the tumor microenvironment following combination treatment with LTX-315 and CAELYX®.

**Fig. 5:**
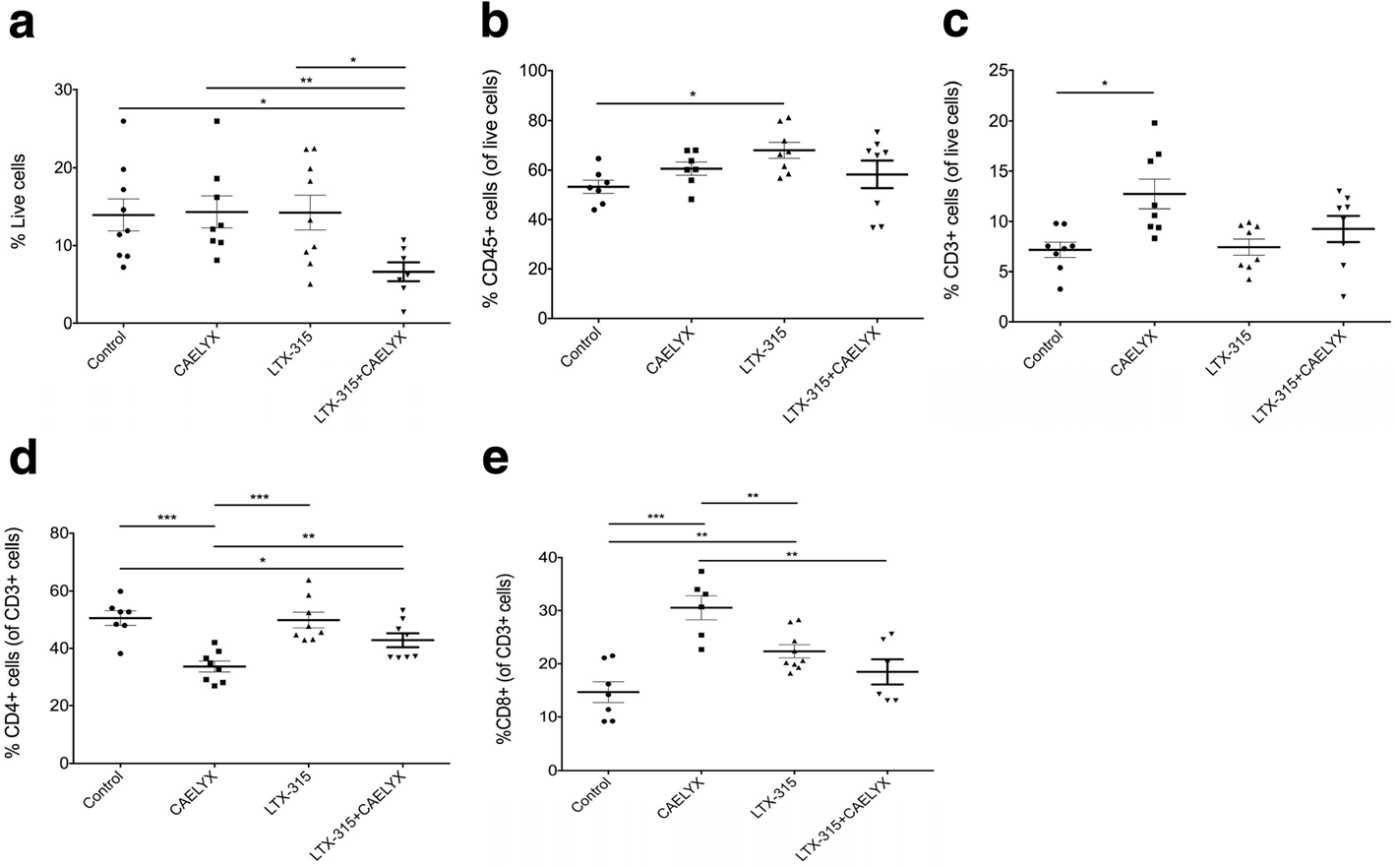
Treatment with CAELYX®, LTX-315, and the combination of both reshapes the tumor microenvironment. Tumors (*n* = 8) were harvested on day 14 (day 7 post treatment) and stained for an array of immune cell markers before being analyzed by flow cytometry. Images show flow cytometry determination of live cells (**a**), CD45+ leukocytes (**b**), CD3+ T lymphocytes (**c**), CD4+ T cells (**d**). and CD8+ cytotoxic T cells (**e**). Each dot represents data for one mouse. Student’s *t* test: *P < 0.05, **P < 0.01, ***P < 0.001

The data from the histological examination were further validated using flow cytometry. Eight animals from each treatment group were treated with LTX-315, CAELYX®, or the combination of both, and analyzed using flow on day 7 post treatment (day 14). In accordance with the histology results, the combination treatment induced very strong necrosis and tumor cell killing as shown by a significantly lower percentage of live cells (Fig. 5a) in the combination group compared to controls and monotherapies. Furthermore, LTX-315 alone induced a significant increase in CD45+ immune cells within the tumor parenchyma compared to control tumors (Fig. 5b). CAELYX® alone also increased the number of CD3+ T cells in the tumor compared to controls (Fig. 5c), while lowering the number of CD4+ T cells in the tumor microenvironment compared to controls, monotherapies, and the combination therapy (Fig. 5d). CAELYX® alone decreased the number of CD4+ T cells inside the tumor parenchyma (Fig. 5d), while CAELYX® and LTX-315 alone induced a significant increase in tumor-infiltrating CD8+ T cells (Fig. 5e). A significant increase in tumor-infiltrating CD8+ T cells was not observed in the combination group.

### Neoadjuvant treatment with LTX-315 in combination with CAELYX® induces complete regression and an increase in overall survival

**Fig. 6:**
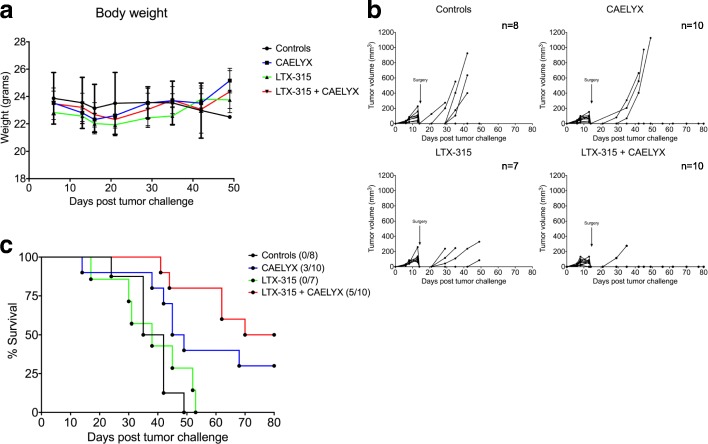
Combination therapy with LTX-315 and CAELYX® induced complete regression of triple-negative breast cancer (TNBC) tumors, and increased survival when used in a neoadjuvant setting. Animals with larger 4 T1 mammary carcinomas (60–100 mm^3^) were divided into four groups, (i) vehicle controls + surgery, (ii) CAELYX® + surgery (iii) LTX-315 + surgery, or (iv) a combination of LTX-315 and CAELYX® + surgery. Palpable tumors were injected intratumorally with 0.5 mg LTX-315 on day 8, 9, and 10 post tumor challenge and 8 mg/kg CAELYX on day 8. Tumors were surgically excised (indicated by arrow) 6 days post treatment. The body weight of all animals remained within the normal range throughout the study (**a**). Tumor growth curves showed that sub-therapeutic doses of LTX-315 in combination with CAELYX® followed by surgery, was able to significantly inhibit larger 4 T1 MFP carcinomas (**b**), and induce complete regression in 50% of the treated animals (**c**), as shown by the Kaplan–Meier survival plot (*p* = 0.0020)

Animals with larger 4 T1 mammary carcinomas were treated with the LTX-315 and CAELYX® combination on day 8 before tumors were surgically excised 6 days later (day 14). LTX-315 was used in a sub-therapeutic setting with intratumoral injections of 0.5 mg peptide on day 8, 9, and 10, with an injection dose equal to 50% of the one used in the first experiment (Fig. 1). The combination of LTX-315 and CAELYX® followed by surgery led to a significant increase in survival compared to monotherapy or vehicle controls and surgery, indicating an additive anticancer effect by the combination compared to monotherapies (Fig. 6). The median survival was 38.5 days in controls, 47 days in the CAELYX® group, 38 days in the LTX-315 group and 75 days in the animals treated with the combination. Complete regression was observed in 50% of animals treated with the combination.

## Discussion

Cancer therapy has been transformed with recent advances in immunotherapy. Nonetheless, further therapeutic advances are needed, with efforts focusing on implementing strategies that combine different cancer treatments. Combination therapy has the potential to dramatically improve the outcome in patients with cancer by harnessing potential synergies that can lead to better clinical responses and increased survival. Over time it is expected that combination strategies will become the standard of care for all cancers, although the discovery of effective combinations is a challenging endeavor [32]. One promising strategy is the combination of immunotherapy and chemotherapy, using immunotherapeutic drugs capable of activating antitumor immunity in combination with chemotherapeutic drugs capable of inducing immunogenic cell death (ICD), while at the same time inhibiting cancer-mediated immunosuppression [29].

Triple-negative breast cancer (TNBC) is recognized as a heterogenous, biologically aggressive disease for which there are few treatment alternatives. There are several promising therapeutic strategies being explored in phase I–III clinical studies, but conventional chemotherapy is still considered the standard of care. Patients with chemo-resistant TNBC have a poor prognosis, and often a very rapid onset of metastasis. However, the highly proliferative status of TNBC also makes it more sensitive to chemotherapy, hence making it a good candidate for combination strategies involving chemotherapy and immunotherapy, since tumors often have an increased immune cell infiltrate [2, 4].

LTX-315 is a de novo designed oncolytic peptide for local treatment of solid tumors, capable of reshaping and reprogramming the tumor microenvironment by ICD [16, 33]. The peptide has a dual mode of action, inducing cancer cell lysis, by destabilizing and disintegrating the cellular membrane, and interacting with intracellular targets such as the mitochondria [18, 20].

To further study the potential of LTX-315 in breast cancer therapy, orthotopical 4 T1 mammary carcinomas were established in syngenic Balb/C mice, and treated with LTX-315 and CAELYX® according to the experimental setup illustrated in Fig. 1. In animals, the 4 T1 cells had highly aggressive properties, forming rapidly growing carcinomas metastasizing to the lungs. All control animals were euthanized before day 30 (Fig. 2b) due to tumor burden or lung metastasis. Sub-therapeutic treatment with LTX-315 did not inhibit tumor growth significantly, whereas CAELYX® alone partially inhibited tumor growth, and resulted in a slight increase in overall survival (Fig. 2b). Thus, the therapeutic efficacy of either monotherapy was limited. Interestingly, when LTX-315 and CAELYX® were used in combination, tumor growth was significantly inhibited and complete tumor regression was achieved in 50% of the animals, indicating a strong additive antitumor effect between LTX-315 and CAELYX® (Fig. 2b), which was verified by statistical analysis of the different treatment groups (*p* ≤ 0.05). In the combination group, 50% of the treated animals were tumor free, as demonstrated by BLI (data not shown) at the end of the study on day 80 (Fig. 2c). Altogether, this suggests a strong and long-lasting antitumor response able to eradicate both local tumor growth and metastasis.

The tumor debulking property of LTX-315 was quantified using BLI and MRI (Fig. 3a and b, respectively). The MRI demonstrated large necrotic areas within the tumor microenvironment on day 14 and 21, both with LTX-315 alone and in the combination group (Fig. 3b). Comparably, CAELYX® alone inhibited tumor growth temporarily, indicating a partial response before recurrence on day 28. CAELYX® alone did not induce significant tumor debulking. LTX-315 in combination with CAELYX® induced complete regression of 4 T1 mammary fat pad carcinomas in several of the treated animals (Fig. 3a and b). Images from BLI exhibited significant differences in signal intensity related to the treatment given, thus a scale bar was not applicable. Treatment with LTX-315 induced local necrosis, leading to leakage and loss of BLI signal, even though tumors recurred later. For this reason, the intensity of the BLI signal did not always reflect the measured tumor size and BLI was used primarily as a method for monitoring metastasis.

To elucidate the mechanism behind the complete regression of tumors treated with the combination, tumor tissue was analyzed by immunohistochemistry (IHC) and flow cytometry. Tumor samples for IHC (*n* = 4) demonstrated that combination therapy with LTX-315 and CAELYX® induced significant necrosis and hemorrhagic damage in the 4 T1 TNBC tumors. In addition, the combination therapy resulted in an increase in tumor-infiltrating CD8+ T cells into the remaining viable tumor tissue on day 6 post treatment. On day 27, a majority of the combination animals were tumor free as illustrated by histological samples showing no remaining viable tumor tissue (Fig. 4).

Flow cytometry (*n* = 8) showed that the combination treatment induced extensive tumor cell killing with very few live cells 7 days post treatment (Fig. 5a). Furthermore, treatment with LTX-315 alone resulted in a significant increase in CD45+ immune cells into the tumor parenchyma. CD45 is expressed on all leukocytes, including neutrophils and natural killer (NK) cells [34]. Given that LTX-315-treatment results in a substantial increase in CD45+ cells compared to the other treatment groups (Fig. 5b), we speculate that immune cells other than CD8+ T cells are also important in the LTX-315-induced antitumor immune response. These may include CD4+ T helper cells and NK cells. Natural killer cells have been shown to have an important role in cancer immunotherapy and are capable of interacting with and killing cancer cells directly by numerous modes of action [35]. Treatment with CAELYX® alone reduced the amount of CD4+ T cells in the tumor microenvironment on day 13 and 14, as demonstrated by both IHC and flow cytometry (Figs. 4 and 5d, respectively). Thus, the additive antitumor effects observed when LTX-315 is combined with CAELYX® may be partially explained by a reduction in immunosuppressive cells, such as CD4+ regulatory T cells (Tregs) and myeloid-derived suppressor cells (MDSCs). Even though little is known about the effect of doxorubicin on Tregs, doxorubicin has been shown to eliminate myeloid-derived suppressor cells (MDSCs) [28]. Moreover, the down-regulation of CD4+ T cells following treatment with CAELYX® alone seems to be a transient process, as demonstrated by an increase in CD4+ T cells on day 27 post treatment (Fig. 4). Treatment with LTX-315 alone and the combination induced an increase in tumor-infiltrating CD4+ T cells on day 13 and 14 (Figs. 4 and 5d). The increase in tumor-infiltrating CD4+ T cells following treatment with LTX-315 alone and the combination, may indicate reshaping of the tumor microenvironment from immunosuppressive CD4+ Tregs to a different CD4+ T cell phenotype important for antitumor immunity. CD4+ T helper cells have indeed been shown to be a critical element in optimal activation of CD8+ T cells and in the maintenance of cancer-related immune memory [36–38].

CAELYX® alone induced significant infiltration of CD8+ cytotoxic T cells compared to the other treatments (Fig. 5e). The increase in tumor-infiltrating CD8+ T cells may be related to the amount of viable tumor tissue left in the treated tumors 7 days post treatment. In the LTX-315 alone and in the combination group most of the tumor tissue was necrotic following treatment, compared to tumors treated with CAELYX® alone, which were less necrotic. In this study, and in previous studies, we have observed that tumor tissue with substantial necrosis following intratumoral treatment does not contain significant numbers of CD8+ T cells in the necrotic region of the tumor, when analyzed by IHC. Accordingly, we observe extensive infiltration of CD8+ T cells residing in the remaining viable tumor tissue. Hence, it is thought that massive necrosis and tumor debulking will lead to a smaller total number of active CD8+ T cells inside the tumor tissue area when analyzed in a quantitative manner.

It is well-established that the location of immune cells is equally important to the total number of immune cells, when it comes to the assessment of immunoscore and patient prognosis and survival [39]. In our study we observed differences in tumor-infiltrating CD4+ and CD8+ T cells following treatment with the combination, when analyzed by IHC and flow (Figs. 4 and 5). Considering IHC is a snapshot of a specific region of the tumor parenchyma, while flow cytometry investigates the total tissue harvested, this is not unexpected. Analysis with IHC was focused on areas of interest, such as the region between the treatment-induced necrosis and the viable tumor tissue. This is the area where CD4+ and CD8+ T cells will exert their cytotoxic antitumor activity as they gain access to viable tumor cells. Contrarily, flow cytometry examines the total tissue harvested and does not differentiate between tumor-excluded or tumor-infiltrating CD4+ and CD8+ T cells. While IHC is a method that evaluates compartmentalization of immune cells, which is very important for immunoscore and clinical response, flow cytometry does not. Thus, it is important to analyze the potential reshaping of the tumor microenvironment in both a qualitative (IHC) and a quantitative (flow cytometry) manner.

Altogether, these data indicate strong reshaping of the tumor microenvironment in treated lesions versus non-treated lesions, thereby resulting in an immune-mediated antitumor response. This reshaping of the tumor microenvironment is dependent on the immunomodulating properties of both LTX-315 and doxorubicin. Conventional chemotherapeutic agents, including doxorubicin, can stimulate the immune system by directly activating CD8+ or γδ T cells, leading to an antitumor T_H1_ response through production of interferon gamma (IFNγ) and IL-17 (reviewed in [40]).

LTX-315 promotes strong local necrosis and ICD through the release of DAMPs, such as ATP, high mobility group box 1 protein (HMGB1) and cytochrome c. DAMPS are important for immune cell activation, antigen presentation, and T cell clonal expansion [16, 18, 40, 41]. This will further induce an intratumoral T cell response shown by an increase in CD3+ tumor-infiltrating lymphocytes (TILs) into the tumor parenchyma [22, 23]. Simultaneously, CAELYX® (doxorubicin) is known to further promote ICD [42] and inhibit the frequency of immunosuppressive cells, such as MDSCs [28]. Furthermore, low-dose metronomic doxorubicin has been shown to inhibit angiogenesis, and inhibit the recruitment of tumor-associated macrophages and cells with tumor-initiating and treatment-resistant properties [43]. Thus, the additive effect between LTX-315 and CAELYX® may arise from the ability of LTX-315 to reshape “cold” tumors into “hot” tumors by stimulating immune activation and an increase in TILs, which is further augmented by doxorubicin’s ability to inhibit angiogenesis, induce T cell infiltration, and deplete immunosuppressive cells [43]. This is also supported by recent evidence demonstrating the importance of TILs in response to therapy and improvement in prognosis in patients with breast cancer [5, 31, 44, 45]. Therefore, the combination of LTX-315 and CAELYX® demonstrates a potential to alter the tumor microenvironment in TNBC by converting it from an immunologically suppressive microenvironment to a more immunologically antitumorigenic microenvironment.

To elucidate the potential of LTX-315 in combination with CAELYX® as a neoadjuvant treatment, 4 T1 breast carcinomas were treated 6 days before tumors were surgically excised from the animals (Fig. 1, experiment 2). At this point, several of the animals already had metastasis, as demonstrated by BLI (data not shown). Most of the animals in this study were euthanized due to metastasis (to the lungs), indicating that a significant increase in survival occurred due to inhibition of metastasis. Our data demonstrate that LTX-315, in combination with CAELYX® and surgery, induced a 60–97% increase in median survival compared to controls or either of the monotherapies and surgery (Fig. 6).

## Conclusions

The mode of action of LTX-315 leads to the release of potent immune stimulators (DAMPs) and tumor antigens, which results in tumor-specific antitumor immune responses. For this reason, LTX-315 is an ideal candidate for combination with therapies capable of removing tumor-induced immune suppression implemented by suppressive cells such as MDSCs and Tregs. The current study verifies that LTX-315 has promising potential to be used in immunochemotherapy, and that such combinations warrant further investigation, aiming for future clinical implementation.

## Abbreviations

BLI: Bioluminescence imaging
CAPs: Cationic antimicrobial peptides
DAMPs: Danger-associated molecular pattern molecules
ER: Estrogen receptor
H&E: Hematoxylin and eosin
HER2: Human epidermal growth factor receptor 2
IL-17: Interleukin 17
INFγ: Interferon gamma
MDSCs: Myeloid derived suppressor cells
MRI: Magnetic resonance imaging
NK: Natural killer
PBS: Phosphate-buffered saline
PR: Progesterone receptor
TILs: Tumor-infiltrating lymphocytes
TNBC: Triple-negative breast cancer
Tregs: Regulatory T cells
